# Efficacy of IVUS-guided stent implantation in patients with complex CAD: a meta-analysis based on RCTs

**DOI:** 10.3389/fcvm.2024.1446014

**Published:** 2024-11-28

**Authors:** Anyi Xu, Dongying Wang, Bangsheng Chen, Siyue Song, Qiufeng Zhang, Zuokun Zhu, Min Dai, Chenyi Wang

**Affiliations:** ^1^ The First Affiliated Hospital of Zhejiang Chinese Medical University (Zhejiang Provincial Hospital of Chinese Medicine), Hangzhou, Zhejiang, China; ^2^ Emergency Medical Center, Ningbo Yinzhou No. 2 Hospital, Ningbo, Zhejiang, China; ^3^ College of Basic Medical Sciences, Zhejiang Chinese Medical University, Zhejiang, China; ^4^ The Second Clinical Medical College, Zhejiang Chinese Medical University, Hangzhou, Zhejiang, China; ^5^ Intensive Care Unit, Ningbo Yinzhou No. 2 Hospital, Ningbo, Zhejiang, China

**Keywords:** IVUS, stent implantation, complex CAD, angiography, meta-analysis

## Abstract

**Background:**

This study is to investigate the efficacy of stent implantation in patients with complex coronary artery disease (CAD) under intravascular ultrasound (IVUS) guidance and non-IVUS guidance.

**Methods:**

We conducted a systematic search in PubMed, Web of Science, Cochran, and Embase for the articles of IVUS-guided and non-IVUS-guided stent implantation in patients with complex CAD and reported related outcomes. We included major adverse cardiovascular events (MACE), myocardial infarction (MI), cardiac death and other outcome indicators. Relative ratio (RR) and 95% confidence interval (CI) were used for statistical analysis.

**Results:**

A total of 5,173 subjects were included in 6 randomized control trials. The results showed that the incidence of MACE (RR: 0.63, 95% CI: 0.49–0.82, *P* < 0.001), cardiac death (RR: 0.61, 95% CI: 0.44–0.85, *P* = 0.004), target vessel revascularization (TVR) (*P* = 0.01), target lesion revascularization (TLR) (*P* = 0.03) and stent thrombosis (ST) (*P* = 0.002) in the experimental group (IVUS-guidance) was lower than that in the control group (non-IVUS-guidance). However, no statistical difference was observed between the both groups in the incidence of MI (*P* = 0.13) and all-cause death (*P* = 0.41).

**Conclusions:**

Compared with the non-IVUS-guided group, IVUS-guided stent implantation may be more effective for patients with complex CAD.

**Systematic Review Registration:**

PROSPERO [CRD42024531366].

## Introduction

Ischemic heart disease is the leading cause of death worldwide, with an annual death toll of about 6.26 million (1). Percutaneous coronary intervention (PCI) is the main interventional therapy for patients with acute coronary syndrome or chronic coronary syndrome who still have symptoms despite receiving drug therapy. Coronary angiography is the most widely used coronary artery disease (CAD) imaging method in the world (2). Angiography is widely used in vascular stent implantation. However, intravascular ultrasound (IVUS) has become an important auxiliary means of angiography due to its limitations such as underestimating the true vascular size, plaque morphology, presence of calcium and thrombus, plaque vulnerability, true lesion length, stent expansion and adherence, residual stenosis after intervention, and presence of dissection (3). Compared with angiography, IVUS has the advantages of providing accurate imaging of vascular size, plaque morphology and dissection, and guiding interventional surgery, including stent size determination, evaluation of residual stenosis and stent attachment and expansion.

Previous meta-analysis has shown that compared with traditional angiography, IVUS has improved the outcome of stent implantation in CAD patients. For example, Fahed Darmoch et al. conducted a meta-analysis and concluded that compared with standard coronary angiography–guided PCI, IVUS imaging-guided optimization of stent implantation is associated with reduced risk of cardiovascular death and major adverse events [such as myocardial infarction (MI), target lesion revascularization (TLR), and stent thrombosis (ST)] (4). After subgroup analysis, Farah Yasmin et al. found that in some outcome indicators [e.g., major adverse cardiovascular events (MACE), ST], no significant difference was observed between the non-complex lesion group, but significant difference was observed between the complex lesion group (2).

Because there is still a lack of meta-analysis to explore the outcome of IVUS-guided stent implantation in patients with complex CAD, this study conducted a meta-analysis to investigate the difference in the efficacy of patients with complex CAD after stent implantation under IVUS guidance and non-IVUS guidance, which was based on published randomized control trials (RCTs).

## Methods

### Literature search

This meta-analysis adheres to the Preferred Reporting Items for Systematic Reviews and Meta-Analyses statement guidelines (2020 edition), and is registered on PROSPERO with the registration mark CRD42024531366. In this study, two independent researchers searched PubMed, Web of Science, Cochran, and Embase for the impact of IVUS-guided stent implantation with or without IVUS-guided stent implantation in patients with complex CAD. If there are differences, the third researcher and the above two researchers will discuss and resolve them. The retrieval time was from the establishment of the database to September 2024, and the PICOS principle is applied. The combination of subject words and free words was used for retrieval, and different retrieval formulas were formulated for different databases. The search formula used is “(complex coronary lesion OR CTO OR chronic total occlusion OR multiple stents) AND (IVUS OR intravascular ultrasound OR intravascular ultrasound-guided OR OCT OR optical coherence tomography OR optical coherence tomography-guided OR angiography OR angiography-guided)”. At the same time, we also manually searched the relevant references mentioned in the original text and the review to avoid omissions. We also searched the Clinicaltrials.gov website to identify articles that are in progress but have not yet published results.

### Inclusion and exclusion criteria

Studies that met the following criteria were included: (1) the study design was a RCT; (2) the subjects were patients with complex CAD; (3) the experimental group had and only had IVUS guidance; (4) the control group was non-IVUS guided; (5) the drug eluting stent (DES) used is second-generation and above; (6) the follow-up time was greater than or equal to 12 months.

Exclusion criteria were as follows: (1) the full text cannot be viewed; (2) the language of the article was not English; (3) the experimental group or the control group had multiple guidance methods; (4) the data cannot be extracted or merged with other data. Only the most comprehensive or latest literature is included.

### Outcomes

PCI is the main interventional therapy for patients with acute coronary syndrome or chronic coronary syndrome who still have symptoms despite receiving drug therapy. The subjects of this meta-analysis were patients with complex CAD, including chronic total occlusion (CTO), long lesions, or the need to implant multiple stents. The outcomes we focused on included, but not limited to, MACE, MI, cardiac death.

### Data extraction and quality evaluation

According to the pre-designed table, the following data were collected for all included articles: authors, publication year, RCT number, trial population area, lesion type, recruitment number, recruitment year, specific treatment method, follow-up time, main outcome of article report, inclusion criteria of each article and definitions of MACE, MI and ST in each article.

The Cochrane bias risk assessment tool in RevMan was used to evaluate the quality of each study in the following seven aspects: (1) random sequence generation (selection bias); (2) allocation concealment (selection bias); (3) blinding of participants and personnel (performance bias); (4) blinding of outcome assessment (detection bias); (5) incomplete outcome data (attrition bias); (6) selective reporting (reporting bias); (7) other bias. According to the actual content of the article, the seven aspects mentioned above were classified into “low risk”, “high risk” and “risk uncertainty”. “High risk” indicates that the possibility of a large discrepancy between the results and the actual intervention results. “Low risk” indicates the opposite. All articles were evaluated by two independent researchers. If there were differences, they were discussed with the third researcher to reach a consensus.

### Statistical analyses

This meta-analysis used Review Manager software (RevMan version 5.3; oxford, U.K.) to combine the collected data. The count data were evaluated by relative risk (RR) and 95% confidence intervals (CI), and the chi-square test was used to evaluate the heterogeneity of statistics. A high degree of heterogeneity was defined when *P* < 0.05. On the contrary, heterogeneity was considered (to be) low. The fixed effect model was used for statistical analysis of the data, and the random effect model was used when the heterogeneity was greater than 50%. All tests were bilateral. Statistical significance was considered when *P* < 0.05.

## Results

### Study selection and characteristics

**Figure 1:**
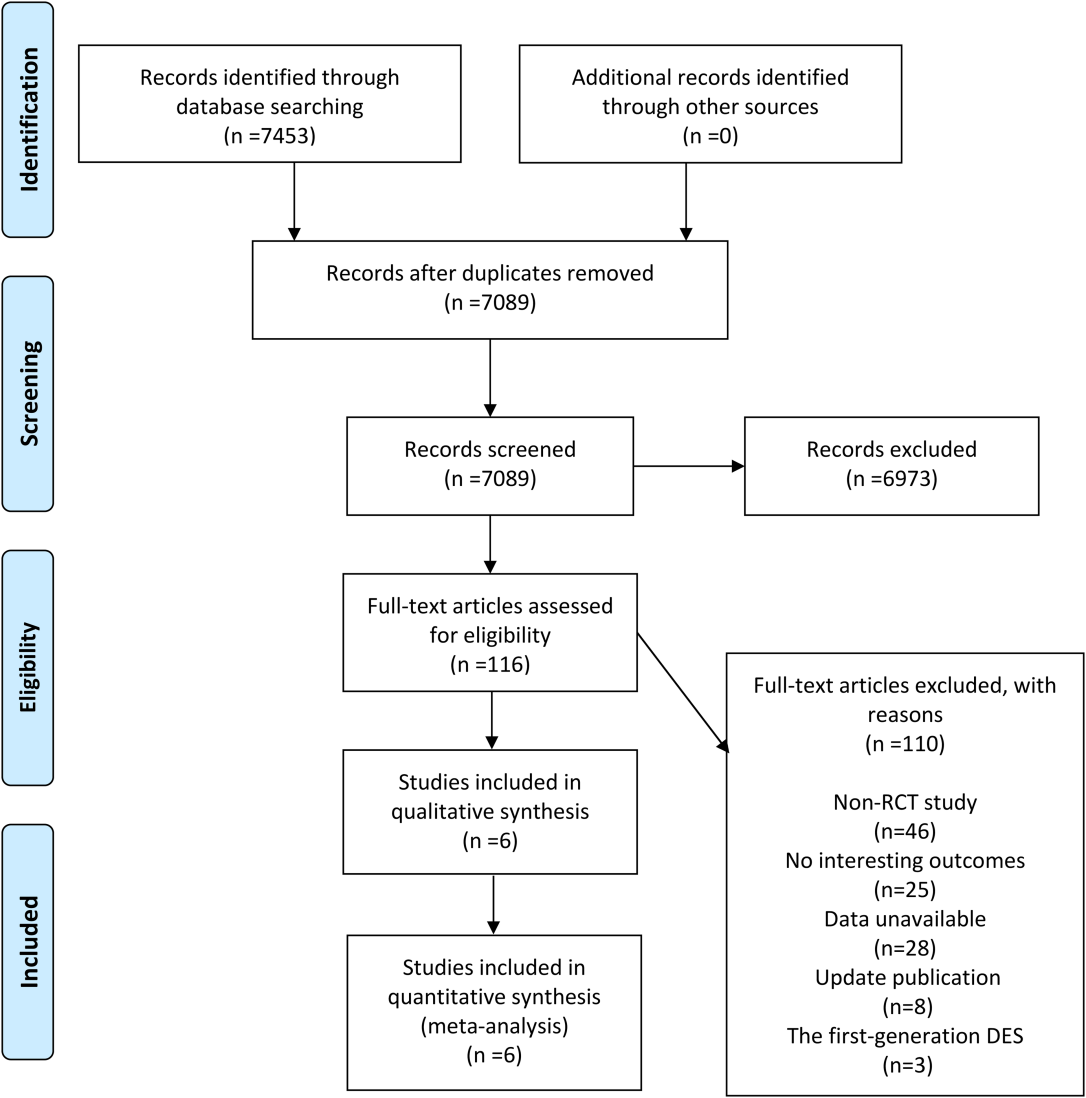
Retrieval and selection process diagram.

After retrieval, a total of 7,453 results were obtained, and 7,089 were left after removing duplicates. After excluding by reading the title and abstract, we finally got 116 articles that needed to read the full text. After screening, 6 articles were finally included in accordance with the inclusion criteria (5–10). The specific retrieval and screening process are shown in Figure 1.

The characteristics of the studies included in this meta-analysis are pooled in Table 1, Supplementary Tables S1, S2. All the included studies were RCTs, with a total of 5,173 patients. All subjects belonged to elective patients. The number of experimental groups in 5 studies was 1:1 compared with the number of control groups, and only one study was 2.5:1. Except that the control group of one study was guided by optical coherence tomography (OCT), the other 5 studies were guided by angiography. The experimental group was all guided by IVUS. There were 1 study with complex lesions, 1 study with CTO lesions, and 2 studies with long lesions, and the remaining two were significant coronary artery lesions and bifurcation lesions. Recruitment years ranged between 2007 and 2018. The subjects were concentrated in South Korea and China. The follow-up time of 5 studies was 12 months, and the remaining 1 study was 25.2 months.

**Table 1:** Characteristics of all the studies included in the meta-analysis.

Author	Year	No. of experimental group	No. of control group	Treatment plan of experimental group	Treatment plan of control group	Lesion type	Follow-up time (mouth)	Patient inclusion criteria
Kim BK (7)	2015	201	201	IVUS	Angiography	CTO lesions	12	Patients with CTO who were aged 20 to 80 years and had typical symptomatic angina or positive test results for functional evaluation of ischemia
Hong SJ (5)	2015	700	700	IVUS	Angiography	Long lesions	12	Patients with typical chest pain or evidences of myocardial ischemia (e.g., stable, unstable angina, silent ischemia and positive functional study or reversible changes in the electrocardiogram consistent with ischemia; stent length ≥28 mm by angiography estimation; significant coronary artery stenosis (>50% by visual estimate) considered for coronary revascularization with stent implantation
Kang D (9)	2023	1,003	1,005	IVUS	OCT	Significant coronary artery lesions	12	Patients ≥19 years of age who were undergoing PCI with contemporary drug-eluting stents or drug-coated balloons (only for in-stent restenosis) for significant coronary artery lesions
Kim JS (8)	2013	269	274	IVUS	Angiography	Long lesions	12	Patients who were over 20 years of age and had a *de novo* lesion requiring a stent ≥28 mm in length in a vessel with a distal reference diameter ≥2.5 mm by visual angiographic estimation
Kwon W (10)	2023	138	54	IVUS	Angiography	Complex lesions	25.2	Having true bifurcation lesion with side branch ≥2.5 mm size, CTO, unprotected left main disease, long coronary lesions, multivessel PCI, multiple stents needed, in-stent restenosis, severely calcified lesions, or coronary ostial lesions
Chen SL (6)	2012	324	304	IVUS	Angiography	Bifurcation lesions	12	/

MI, myocardial infarction; CTO, chronic total occlusion; PCI, percutaneous coronary intervention; OCT, optical coherence tomography; IVUS, intravascular ultrasound; DES, drug-eluting stents.

### Risk of bias assessment

Most of the literature was rated as low to medium risk, and 2 articles had a high risk of attrition bias (5, 8). The main reason was that more data were lost during the follow-up process or the explanation of some missing data was not clear. The attrition bias of the two studies was unknown risk (9, 10) because the process of collecting data was not described in detail. The selection bias of the two studies was unknown risk (7, 8), mainly due to the unclear description of the recruitment conditions of the subjects. Other biases in the two studies were unknown risk (5, 6) because there were some factors that could lead to other biases. The complete bias risk assessment results are shown in Supplementary Figures S1, S2.

### Analysis of main outcome indicators

Outcome measures included: MACE, MI, cardiac death, all-cause death, ST, TLR, target vessel revascularization (TVR). Among them, subgroup analysis was performed on MACE and cardiac death groups.

**Figure 2:**
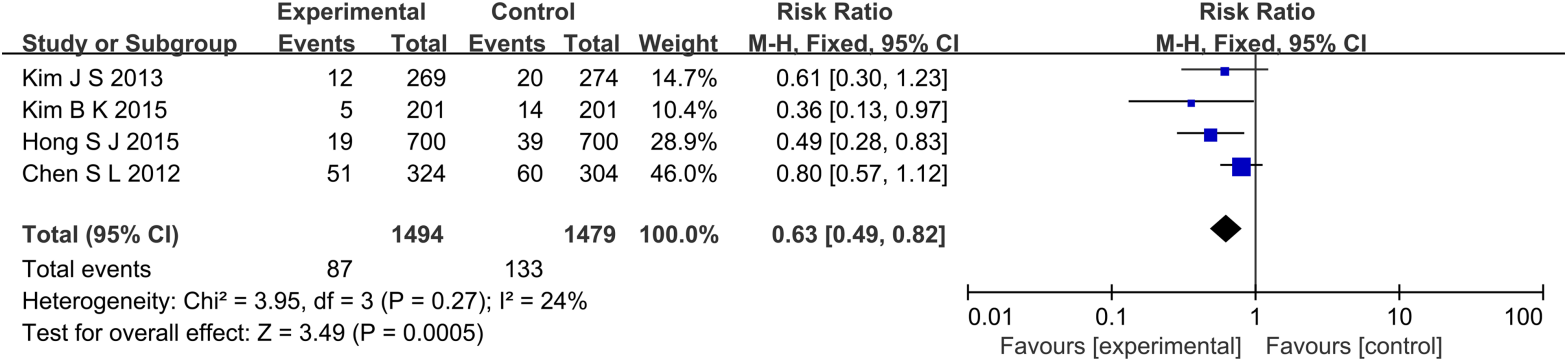
The forest plot of the effect of IVUS-guided and non-IVUS-guided stent implantation in patients with complex CAD on the incidence of MACE.

**Figure 3:**
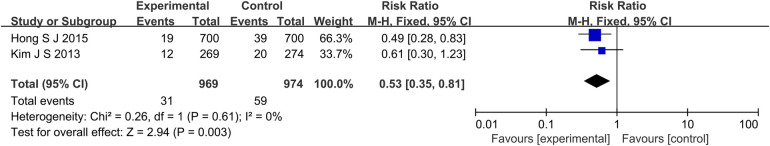
The forest plot of the effect of IVUS-guided and non-IVUS-guided stent implantation in patients (long lesions) with complex CAD on the incidence of MACE.

A total of 4 studies reported MACE outcome indicators (Figure 2), a total of 2,973 subjects were reported. The combined results showed that the incidence of MACE in the experimental group was lower than that in the control group (RR: 0.63, 95% CI: 0.49–0.82, *P* < 0.001, *I*^2^ = 24%, fixed effect model). Subgroup analysis showed that for patients with long lesions, the experimental group had a lower incidence of MACE (RR: 0.53, 95% CI: 0.35–0.81, *P* = 0.003, *I*^2^ = 0%, fixed effect model) (Figure 3).

**Figure 4:**
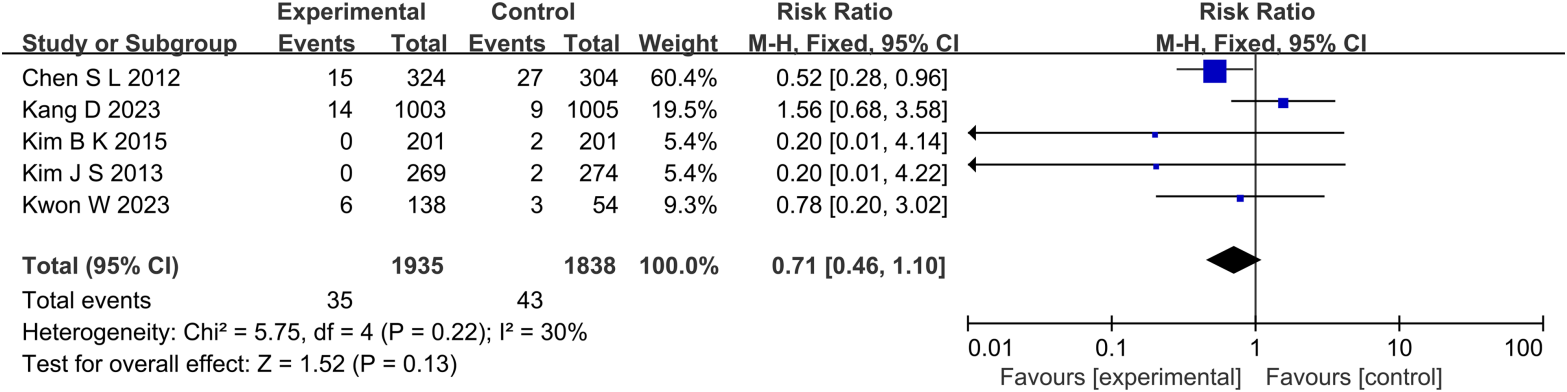
The forest plot of the effect of IVUS-guided and non-IVUS-guided stent implantation in patients with complex CAD on the incidence of MI.

A total of 5 studies reported the outcome indicators of MI (Figure 4), with a total of 3,773 subjects. Statistics showed that there was no significant difference in the incidence of MI between the experimental group and the control group (RR: 0.71, 95% CI: 0.46–1.10, *P* = 0.13, *I*^2^ = 30%, fixed effect model).

**Figure 5:**
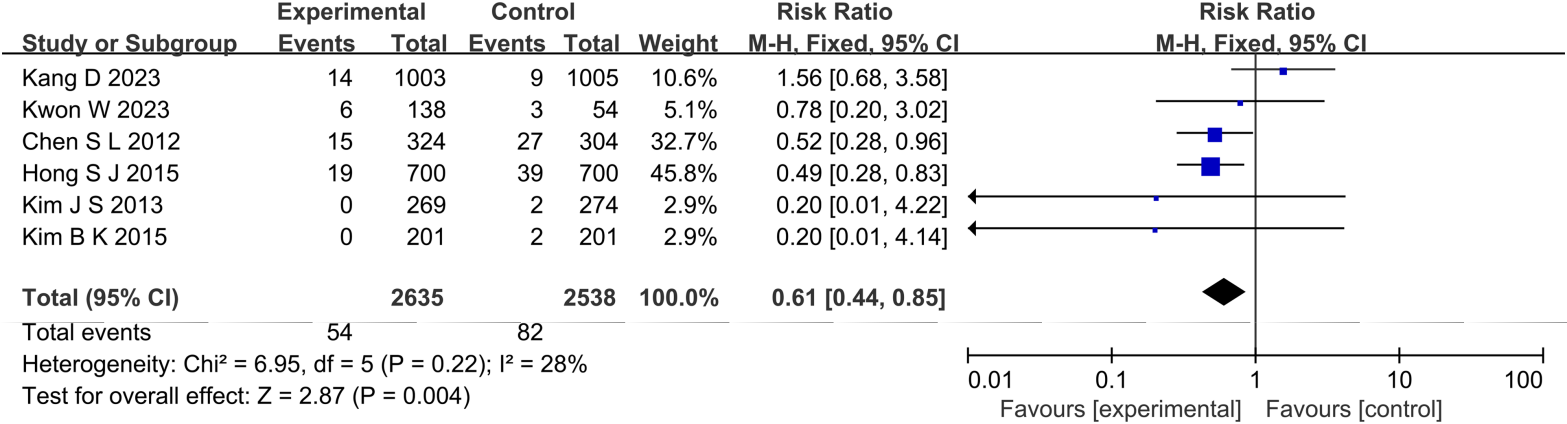
The forest plot of the effect of IVUS-guided and non-IVUS-guided stent implantation in patients with complex CAD on the incidence of cardiac death.

**Figure 6:**
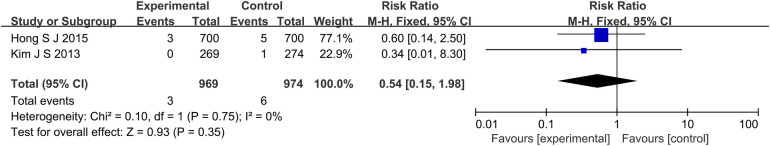
The forest plot of the effect of IVUS-guided and non-IVUS-guided stent implantation in patients (long lesions) with complex CAD on the incidence of cardiac death.

**Figure 7:**
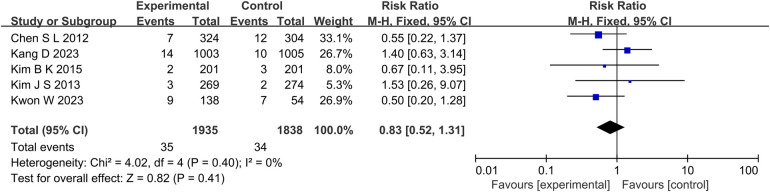
The forest plot of the effect of IVUS-guided and non-IVUS-guided stent implantation in patients with complex CAD on the incidence of all-cause death.

The results of 6 studies showed that in terms of cardiac death (Figure 5), the incidence of cardiac death in the experimental group was lower than that in the control group (RR: 0.61, 95% CI: 0.44–0.85, *P* = 0.004, *I*^2^ = 28%, fixed effect model). Subgroup analysis showed that for patients with long lesions (*P* = 0.35, *I*^2^ = 0%, fixed effect model), there was no significant difference in the incidence of cardiac death between the both groups (Figure 6). Similarly, there was also no significant difference in the incidence of all-cause death between the experimental group and the control group (*P* = 0.41, *I*^2^ = 0%, fixed effect model) (Figure 7).

**Figure 8:**
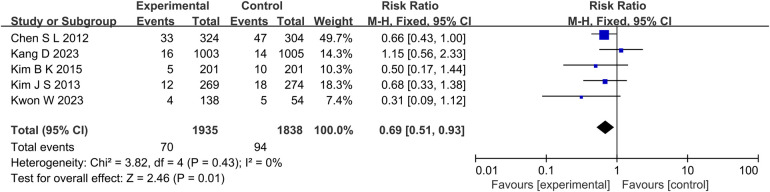
The forest plot of the effect of IVUS-guided and non-IVUS-guided stent implantation in patients with complex CAD on the incidence of TVR.

**Figure 9:**
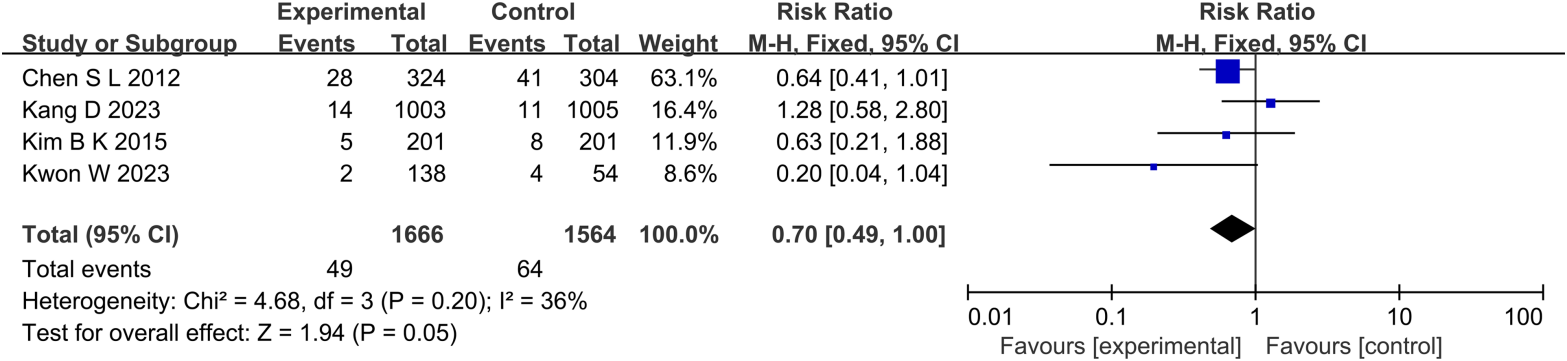
The forest plot of the effect of IVUS-guided and non-IVUS-guided stent implantation in patients with complex CAD on the incidence of TLR.

**Figure 10:**
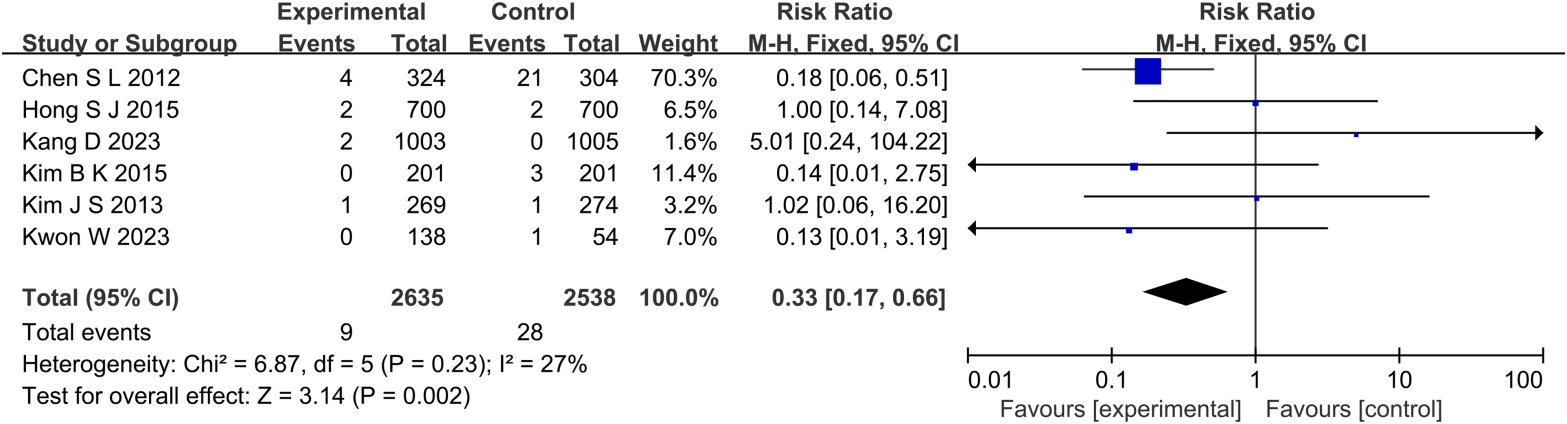
The forest plot of the effect of IVUS-guided and non-IVUS-guided stent implantation in patients with complex CAD on the incidence of ST.

At the same time, in the TVR aspect (*P* = 0.01, *I*^2^ = 0%, fixed effect model) (Figure 8), TLR aspect (*P* = 0.05, *I*^2^ = 36%, fixed effect model) (Figure 9), ST aspect (*P* = 0.002, *I*^2^ = 27%, fixed effect model) (Figure 10), the incidence of related outcomes in the experimental group was lower than that in the control group.

## Discussion

Due to the wide application of PCI in ischemic heart disease, various guidance methods have developed rapidly, and the efficacy of different guidance methods for patients with different conditions is often of concern. Therefore, it is very important and necessary to conduct a meta-analysis of the efficacy of stent implantation in patients with complex CAD under IVUS guidance and non-IVUS guidance.

A total of 6 randomized controlled trials involving 5,173 patients were included in this study. MACE, MI, cardiac death, all-cause death, ST, TLR, TVR, a total of 7 common outcome indicators after PCI were studied. In addition, subgroup analysis was performed on the lesion type of MACE and cardiac death groups. The results showed that the overall incidence of MACE in the experimental group was lower than that in the control group (including in patients with long lesions). In the overall incidence of MI, no significant difference was observed between the experimental group and the control group. The overall incidence of cardiac death in the experimental group was lower than that in the control group. In the subgroup analysis, no significant difference was observed in the patients with long lesions. Similarly, no significant difference in the incidence of all-cause death was observed between the both groups. In TVR, TLR and ST aspects, the incidence of the experimental group was lower than the control group.

MACE is the main factor of adverse clinical outcomes after surgery, and PCI is no exception (11). Compared with angiography, IVUS guidance can reduce the incidence of postoperative MACE in patients with complex CAD, which may be attributed to the following points: (1) the minimum lumen diameter after IVUS guidance is larger, (2) IVUS-guided assisted post-dilatation can be performed more frequently with larger balloons (5), (3) IVUS provides more detailed information on lesion specificity, vascular anatomical features, immediate complications, etc., and can avoid the use of too small stents (6).

In clinical practice, the above mechanism is manifested as a decrease in the incidence of ST, TLR and TVR in patients with complex CAD. Shao-Liang Chen et al. pointed out that IVUS reduces the probability of ST by avoiding the use of too small stents (6). Probal Roy et al. found that ST was significantly reduced in patients receiving IVUS guidance, and the incidence of TLR tended to decrease (12). Gianni Casella mentioned in the article that most trials showed a 38% reduction in the incidence of TVR observed under IVUS guidance (13). Some scholars have pointed out that ST was catastrophic for PCI. Although it is not common, it occupies a central position in the risk-benefit equation of PCI (14). Sung-Jin Hong et al. pointed out that IVUS-guided stent implantation was associated with a significant absolute reduction of 2.9% and a relative reduction of 48% in the incidence of MACE at 1 year of follow-up, and these differences were mainly due to the reduction of TLR in the IVUS-guided group (5).

In the study included in this paper, the incidence of MACE in the experimental group was lower than that in the control group, which was consistent with the results of the above scholars. We speculate that the decrease in the incidence of MACE in the experimental group may be due to the decrease in the incidence of ST, TLR and TVR after surgery. In order to further explore the effect of IVUS-guided stent implantation, this article also performed an additional subgroup analysis of the lesion type of the MACE group. In terms of lesion type, the incidence of MACE in the experimental group was lower than that in the control group in patients with long lesions. We believe that it may be because patients with long lesions can lead to more auxiliary balloon filling, thereby improving the minimum lumen diameter. In summary, after IVUS-guided stent implantation for patients with complex coronary artery lesions, there are advantages in MACE, including patients with long lesions.

More than half of cardiovascular deaths are caused by acute MI. About 550, 000 cases of acute MI first attack and 200,000 cases of recurrence occur every year. Therefore, MI is also the main outcome of people's attention after PCI. Compared with angiography, IVUS guidance can provide more accurate lumen and vascular size, so IVUS is more repeatable and accurate than angiography in evaluating diseases. However, for MI, Helen Parise and other scholars pointed out that although IVUS guidance may bring a weak advantage to MI, this weak advantage may be offset by the increase in the incidence of perioperative MI caused by stent implantation. Therefore, it is not surprising that stent implantation with different guidance methods has no statistical difference in the incidence of MI (15). In the study included in this article, there was no significant difference in the incidence of MI between the experimental group and the control group, which was consistent with the situation mentioned above.

It is estimated that about 40%–50% of cardiovascular deaths are caused by sudden cardiac death, while the global survival rate of cardiac arrest is less than 1% (16). Therefore, it is necessary to pay attention to the cardiac death after PCI. In the study included in this article, the incidence of cardiac death in the experimental group was lower than that in the control group. In addition to the higher accuracy and larger minimum lumen diameter of the IVUS guidance mentioned above, it was also related to the IVUS optimization of stent expansion pointed out by Helen Parise and other scholars to reduce restenosis and repeated revascularization (15). After subgroup analysis, no significant difference was observed between the experimental group and the control group when the lesion type was long. We speculate that it is due to the poor basic conditions of patients with long lesions and more complications. In summary, IVUS-guided stent implantation for patients with complex coronary artery lesions can reduce the cardiac mortality of patients.

Similarly, all-cause death is also one of the outcomes of PCI. In the studies included in this article, no significant difference was observed between the experimental group and the control group for all-cause death. This may be due to the weak advantage of IVUS is not enough to improve the remaining complications of patients, especially some dangerous complications with higher mortality. Therefore, there was no significant difference in all-cause death between IVUS-guided and non-IVUS-guided stent implantation in patients with complex CAD.

## Advantages and disadvantages

This article is the first meta-analysis of the efficacy of stent implantation in patients with complex CAD under IVUS guidance and non-IVUS guidance. A more comprehensive analysis of the common outcomes that may occur after stent implantation in patients with complex coronary artery lesions under IVUS guidance provides some reliable evidence for its application. The studies included in this article are all high-quality RCTs, and the DES types used are all second-generation or above. The limitation of this article is that due to the limited experimental data, it is impossible to perform subgroup analysis on more results. In some subgroup analyses, there are some contingencies in the data processing due to the small number of experiments. At the same time, some of the included studies have a high selection bias, which affects the generalization of this article.

## Conclusion

This meta-analysis showed that IVUS guidance significantly reduced the incidence of MACE, cardiac death, TVR, TLR, and ST in patients with complex CAD after stent implantation compared with non-IVUS guidance. There was no significant difference in the incidence of MI between IVUS-guided and non-IVUS-guided. This shows that IVUS-guided stent implantation in patients with complex CAD has certain advantages, but more RCTs are needed to verify it.

## Data Availability

The original contributions presented in the study are included in the article/Supplementary Material, further inquiries can be directed to the corresponding author.

## Supplementary material

Supplementary Figure 1 Risk of bias graph of the included RCTs.

Supplementary Figure 2 Risk of bias summary of the included RCTs.

Supplementary Table 1 Characteristics of all the studies included in the meta-analysis.

Supplementary Table 2 The definitions of MACE, MI, and cardiac death in the all studies included in the meta-analysis.
