# Supplementary figures and images for: Efficacy of IVUS-guided stent implantation in patients with complex CAD: a meta-analysis based on RCTs
Source: Front Cardiovasc Med. 2024 Nov 28;11:1446014. doi: 10.3389/fcvm.2024.1446014 (PMC11634805; doi:10.3389/fcvm.2024.1446014)

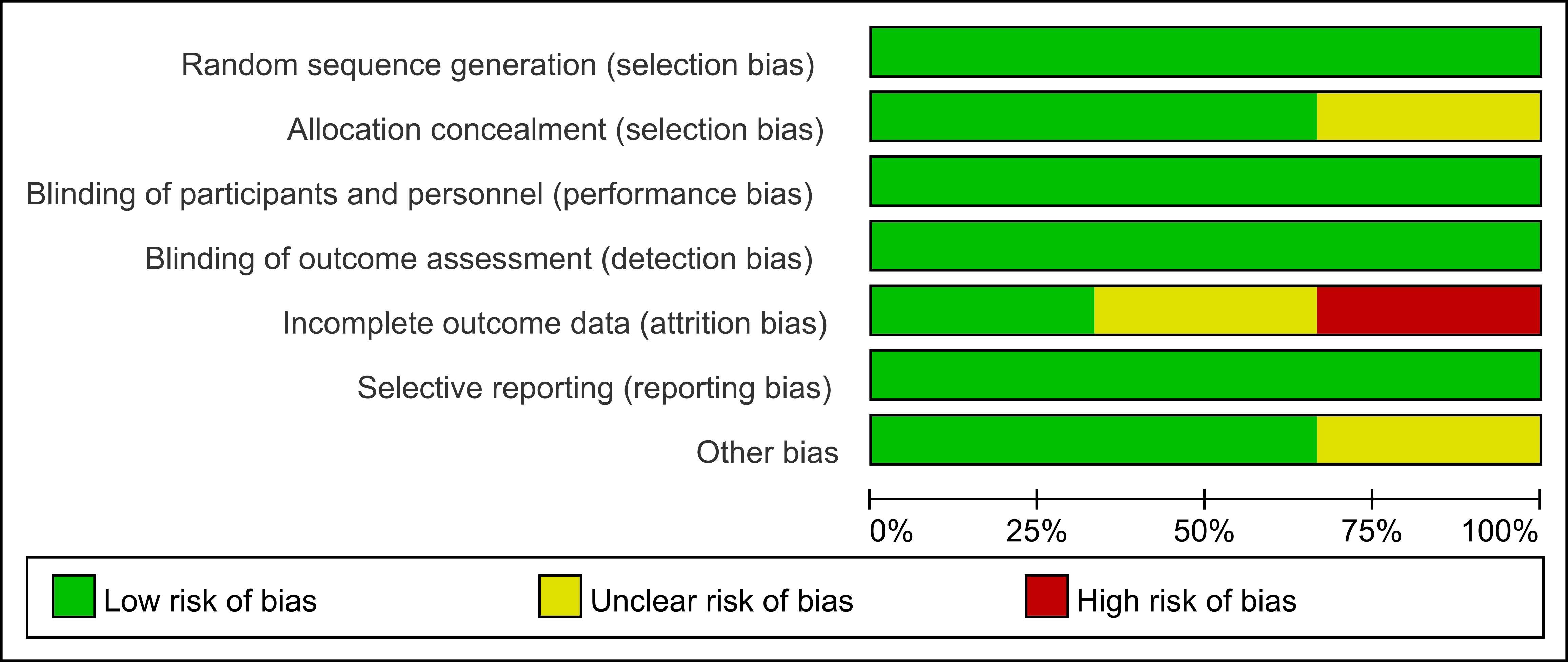

Supplement: Supplementary Figure 1 — Risk of bias graph of the included RCTs. [file Image1.tif]

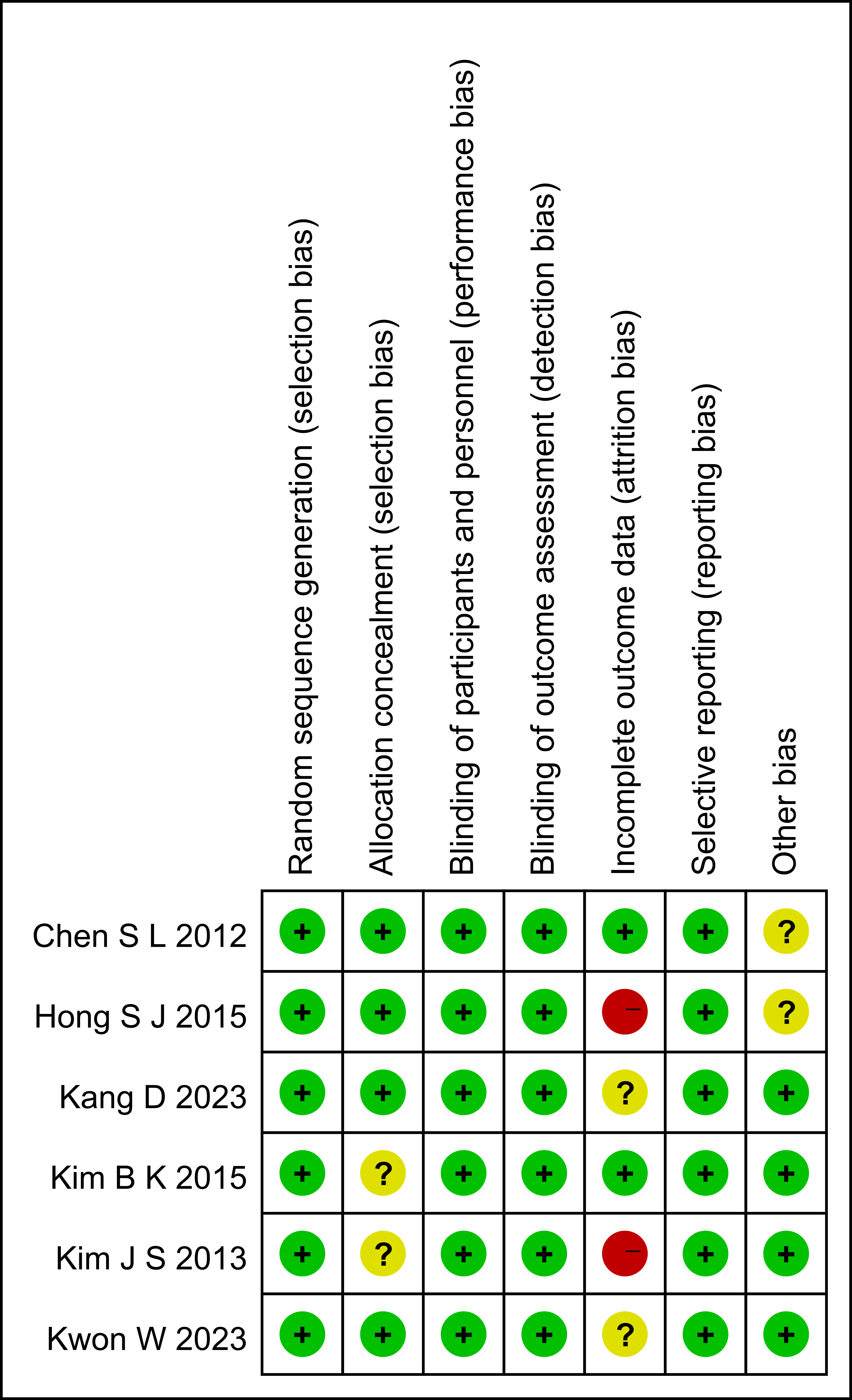

Supplement: Supplementary Figure 2 — Risk of bias summary of the included RCTs. [file Image2.tif]
