# Supplementary material for: Efficacy of IVUS-guided stent implantation in patients with complex CAD: a meta-analysis based on RCTs
Source: Front Cardiovasc Med. 2024 Nov 28;11:1446014. doi: 10.3389/fcvm.2024.1446014 (PMC11634805; doi:10.3389/fcvm.2024.1446014)
Supplement: Supplementary Table 1 — Characteristics of all the studies included in the meta-analysis. [file Table1.docx]

**Supplementary Table 1. Characteristics of all the studies included in the meta-analysis.**

| Author | Year | NCT number | Region of the test population | Recruitment year | Lesion length of experimental group (mean, mm) | Lesion length of control group (mean, mm) | Main outcome |
| --- | --- | --- | --- | --- | --- | --- | --- |
| Kim B K | 2015 | 01563952 | Korea | 2012 | 36.3 | 35.5 | cardiac death and a MACE defined as the composite of cardiac death, MI, or TVR,  respectively |
| Hong S J | 2015 | 01308281 | Korea | 2011 | 34.7 | 35.2 | the composite of MACEs, including cardiac death, target lesion-related MI, or  ischemia-driven TLR at 1 year |
| Kang D | 2023 | 03394079 | Korea | 2018 | 47.8 | 47.2 | a composite of death from cardiac causes, target vessel–related MI, or ischemia-driven TVR at 1 year |
| Kim J S | 2013 | 01145079 | Korea | 2009 | 29.8 | 30.5 | a composite of MACE, including cardiovascular death, MI, TVR, or ST at 1 year following intervention |
| Kwon W | 2023 | 03381872 | Korea | 2017 | 25.0 | 22.1 | target vessel failure defined as a composite of cardiac death, target vessel–related MI, or clinically driven TVR |
| Chen S L | 2012 | / | China | 2007 | 32.67 | 30.53 | MACE, including cardiac death, ST, MI and TLR/TVR |

MI, myocardial infarction; MACE, major adverse cardiac events; ST, stent thrombosis; TLR, target lesion revascularization; TVR, target vessel revascularization.
