# Supplementary material for: Efficacy of IVUS-guided stent implantation in patients with complex CAD: a meta-analysis based on RCTs
Source: Front Cardiovasc Med. 2024 Nov 28;11:1446014. doi: 10.3389/fcvm.2024.1446014 (PMC11634805; doi:10.3389/fcvm.2024.1446014)
Supplement: Supplementary Table 2 — The definitions of MACE, MI, and cardiac death in the all studies included in the meta-analysis. [file Table2.docx]

**Supplementary Table 2. The definition of MACE, MI and ST of all the studies included in the meta-analysis.**

| Author | Year | Definition of MACE | Definition of MI | Definition of ST |
| --- | --- | --- | --- | --- |
| Kim B K | 2015 | Defined as the composite of cardiac death, MI, or TVR at 12 months. | MI was defined as the presence of clinical symptoms, electrocardiographic changes, or abnormal imaging findings associated with MI combined with an increase in creatine kinase-MB above the upper normal limit or troponin T/I greater than the 99th percentile of the upper normal limit, unrelated to an interventional procedure. | ST was defined as definite or probable ST according to the Academic Research Consortium. |
| Hong S J | 2015 | Including cardiac death, target lesion–related MI, or ischemia-driven TLR at 1 year. | Target lesion–related MI during the 1-year follow-up after hospital discharge was defined as the presence of clinical symptoms, electrocardiographic changes, or abnormal imaging findings of MI, combined with an increase in the creatine kinase MB fraction above the upper normal limits or an increase in troponin T or troponin I to a level greater than the 99th percentile of the upper normal limit. The territory of the MI was supplied by the coronary artery containing the stented lesions (implanted stent≥28 mm in length). | Definite, probable, and possible ST was defined according to the recommendations of the Academic Research Consortium. |
| Kang D | 2023 | / | / | / |
| Kim J S | 2013 | Including cardiovascular death, MI, ST, or TVR at 1 year after procedure. | MI was defined as the presence of clinical symptoms, electrocardiographic changes, or abnormal imaging findings of MI combined with an increase in creatine kinase myocardial band fraction to greater than 3× the upper limit of the normal range or an increase in troponin T/troponin I to more than the 99th percentile of the upper normal limit, unrelated to an interventional procedure. | Definite, probable, and possible ST was defined according to the recommendations of the Academic Research Consortium. The timing of ST was classified as acute (within 24h), subacute (1 day to 1 month), and late (from day 31 to 365) post-index procedure. |
| Kwon W | 2023 | / | The definition of MI used in this trial was based on the third universal definition for spontaneous MI, and the Society for Cardiovascular Angiography and Interventions definition for procedure-related MI. | / |
| Chen S L | 2012 | Including cardiac death, MI, or TVR. | MI was diagnosed if the plasma level of CK-MB increased to >1 times the prevalue immediately before stenting in patients with AMI. | ST were defined according to guidelines set by the Academic Research Consortium. |

MI, myocardial infarction; MACE, major adverse cardiac events; ST, stent thrombosis; TLR, target lesion revascularization; TVR, target vessel revascularization; AMI,

acute myocardial infarction.
